# The Albany Hospital and the Blaauwkrantz Accident

**Published:** 1911-06-17

**Authors:** 


### THE ALBANY HOSPITAL AND THE BLAAUWKRANTZ ACCIDENT.

### SECRETARY'S ACCOUNT OF THE ARRIVAL OF THE INJURED.

So much sympathy was excited in England at the appalling railway disaster which on Saturday, April 24 last, caused the death of 30 persons, by the falling of a passenger train from the Blaauwkrantz Bridge, on its way from Port Alfred to Grahamstown, Cape Colony, that the following account from the Secretary of the hospital which organised the work of rescue will be read with interest. The bridge, which is of one span, and generally regarded as the most graceful in the Colony, crosses a chasm some 250 feet deep. Mr. Arthur Jubb, Secretary of the Albany Hospital, Grahamstown, has sent us a full account. The way in which the hospital at a moment's notice rose to the occasion, and provided beds for an unknown number of injured, is simply told in his own words.

"The violent ringing of the telephone bell was the first announcement at the hospital that something was wrong somewhere. A peremptory message for the attendance of the House Surgeon or the Matron at the instrument with the least delay possible betokened something serious. The message was enough to alarm the steadiest of matrons, and in this case enough to cause the greatest concern. 'Train gone over Blaauwkrantz Bridge; extent of calamity not known; prepare for the worst; send what help you can.' Immediately the House Surgeon decided to go to the scene of the accident, and, picking two nurses from the staff, set to work gathering together such necessaries in splints and drugs and plasters, as were sure to be wanted. The first business was, of course, to get off this party as speedily as possible, and the whole nursing staff concentrated their efforts upon this task. The relief train was to start in an hour, with such medical service as was available, and every one worked with a zest, touched with the keenest anxiety.

"The doctor and his helpers were soon off to the station. After their departure, attention was given to possible requirements; wards were secured, in which beds were promptly made, linen and towels were requisitioned from the town, and everything was put in a state of preparedness for the arrival of the injured. Owing to the telegraph wires being broken, no news that could be relied on was forthcoming, but it was known that a telegraphist had gone down to tap the wire and send on information. There was nothing to do but to wait, and to see in the interim that everything was at hand that was likely to be wanted. It was quite two in the afternoon (the wrecked train had been due at Grahamstown at 10.20) before the whistle of the train was heard, and very soon after there began to arrive, one after the other, the suffering victims of parsimonious railway mismanagement. At the station ladies and friends had combined to give first attention, and a number of impromptu stretchers had been made out of any available material. Roughly made, they were of the utmost service in conveying the injured to the hospital, where they were received and drafted off to the wards under the supervision of the medical staff, who were there in readiness to render more effective service. The nature of the injuries was as expected. Broken limbs, severe concussions and abrasions. Some were senseless, others jabbered like idiots, but all were seriously damaged. How any one came out alive from that fall of a clear 178 feet on to the rocks below is a mystery. One was a youth with a neck as nearly broken as it could be, and yet conscious; another, a child with its head dented in such a way that an operation seemed inevitable, but, though no operation was done, the recovery in this case has been grand. There was a young woman with both jaws broken, shoulder almost cut off, and both thighs broken, which was one of the most pitiful cases. Only the day before she had been at a wedding, and yet at the time of writing, though there is evidence of internal injury, she is alive and progressing favourably. Broken legs and arms, scalp wounds, dislocated shoulders and crushed feet, were attended to as quickly as possible, the sufferers soothed and quieted, till when the shades of evening descended, all had been done for the alleviation of pain and for the comfort of the wounded that it was possible to do.

"The incoming of twenty-two extra surgical cases into a hospital within a few hours would be a severe tax upon its staff and its resources, but with some thirty other patients in hospital at the time, requiring more or less care and attention, it is a matter for congratulation that a most effective service was rendered by the nursing staff, which consists of three staff nurses and twelve probationers, and two male nurses, who were quite equal to the occasion. No one was flurried or lost his head. The voice of the doctors had a steadying effect upon all, and though to some of the younger ones this unusual sight of extensive bodily injuries must have been very trying to their nerves, yet no one failed or faltered in carrying out the instructions issued. Too much cannot be said in praise of the two nurses who went down to the accident and worked hard under most trying circumstances. To see the place from which the injured had to be got, in many instances extricated from the débris and carried up the steep rocks till they could be put down on some bit of level to be attended to, is to realise that it was no small task.

"Our hospital is not one in which surgical cases are numerous, hence it is all the more a wonder that the staff proved its sterling worth under exceptional stress. So far there have been three deaths, but it is expected that the rest will recover, though some will be maimed for life."

It will be remembered that the Albany General Hospital has eighty beds, and, according to the latest returns available, treats some four hundred in-patients every year. It was founded in 1857.

